# A Distinctive Metabolomics Profile and Potential Biomarkers for Very Long Acylcarnitine Dehydrogenase Deficiency (VLCADD) Diagnosis in Newborns

**DOI:** 10.3390/metabo13060725

**Published:** 2023-06-05

**Authors:** Rajaa Sebaa, Reem H. AlMalki, Wafaa Alseraty, Anas M. Abdel Rahman

**Affiliations:** 1 Department of Medical Laboratories, College of Applied Medical Sciences, Shaqra University, Al-Dawadmi 17472, Saudi Arabia; 2 Metabolomics Section, Department of Clinical Genomics, Center for Genomics Medicine, King Faisal Specialist Hospital and Research Centre (KFSHRC), Riyadh 11211, Saudi Arabia; 3 Department of Nursing, College of Applied Medical Sciences, Shaqra University, Al-Dawadmi 17472, Saudi Arabia; 4 Department of Biochemistry and Molecular Medicine, College of Medicine, Al Faisal University, Riyadh 11533, Saudi Arabia

**Keywords:** VLCADD, newborns, untargeted metabolomics profiling, potential biomarkers

## Abstract

Very long-chain acylcarnitine dehydrogenase deficiency (VLCADD) is a rare inherited metabolic disorder associated with fatty acid *β*-oxidation and characterized by genetic mutations in the *ACADVL* gene and accumulations of acylcarnitines. VLCADD, developed in neonates or later adults, can be diagnosed using newborn bloodspot screening (NBS) or genetic sequencing. These techniques have limitations, such as a high false discovery rate and variants of uncertain significance (VUS). As a result, an extra diagnostic tool is needed to deliver improved performance and health outcomes. As VLCADD is linked with metabolic disturbance, we postulated that newborn patients with VLCADD could display a distinct metabolomics pattern compared to healthy newborns and other disorders. Herein, we applied an untargeted metabolomics approach using liquid chromatography–high resolution mass spectrometry (LC-HRMS) to measure the global metabolites in dried blood spot (DBS) cards collected from VLCADD newborns (*n* = 15) and healthy controls (*n* = 15). Two hundred and six significantly dysregulated endogenous metabolites were identified in VLCADD, in contrast to healthy newborns. Fifty-eight and one hundred and eight up- and down-regulated endogenous metabolites were involved in several pathways such as tryptophan biosynthesis, aminoacyl-tRNA biosynthesis, amino sugar and nucleotide sugar metabolism, pyrimidine metabolism and pantothenate, and CoA biosynthesis. Furthermore, biomarker analyses identified 3,4-Dihydroxytetradecanoylcarnitine (AUC = 1), PIP (20:1)/PGF1alpha) (AUC = 0.982), and PIP2 (16:0/22:3) (AUC = 0.978) as potential metabolic biomarkers for VLCADD diagnosis. Our findings showed that compared to healthy newborns, VLCAADD newborns exhibit a distinctive metabolic profile, and identified potential biomarkers that can be used for early diagnosis, which improves the identification of the affected patients earlier. This allows for the timely administration of proper treatments, leading to improved health. However, further studies with large independent cohorts of VLCADD patients with different ages and phenotypes need to be studied to validate our potential diagnostic biomarkers and their specificity and accuracy during early life.

## 1. Introduction

Long-chain fatty acids (LCFA) play a crucial role as vital energy sources for various body tissues and organs, such as the liver, heart, and skeletal muscle, enabling them to carry out their essential functions and ensure survival. Specifically, LCFAs are transported into the mitochondrial matrix via the carnitine shuttle [1]. After that, fatty acids undergo mitochondrial fatty acid *β*-oxidation to be metabolically oxidized via very long-chain acyl-CoA dehydrogenase (VLCAD). The last is a mitochondrial enzyme, essentially required for the first step of the oxidation of long-chain fatty acyl-CoAs with a chain length of 12–20 carbons [2]. The responsible enzyme for the breakdown of very long-chain fatty acids is produced by a nuclear gene called acyl-CoA dehydrogenase very long chain (*ACADVL*). Mutations in this gene have been detected in newborn patients during the early stages of life. This is linked to a condition known as very long-chain acyl-CoA dehydrogenase deficiency (VLCADD), which falls under the category of inherited metabolic disorders (IMDs) [3]. VLCADD is associated with elevated levels of long-free fatty acids, complex lipids, and acylcarnitines. Lastly, the main biomarker of VLCADD patients specifically is characterized by toxic accumulations of C14:1-carnitine, C14:2-carnitine, C14:1/C16-carnitine ratio, C14:1/C2-carnitine ratio, and C14:1/C12:1 ratio [4,5,6]. These abnormal accumulations of acylcarnitines can negatively affect the physiology of tissues and organs, leading to clinical complications that could be life-threatening and lethal [7]. To illustrate, the elevated toxic accumulation of acylcarnitines causes increased cellular permeability, releasing intracellular proteins and altering cellular function.

The cellular inflammation is increased in VLCADD, where insulin-stimulated glucose uptake is decreased, via Ca^+2^-mediated pathways. Also, VLCADD causes lipid toxicity and cellular stress, subsequently leading to cellular death [8,9,10,11]. The manifestations of VLCADD in patients can develop during the neonatal period or adulthood, and include hypoglycemia, hypoketotic, lactic acidemia, hyperammonemia, cardiomyopathy, and rhabdomyolysis [12,13]. Recently, there have been newly reported manifestations observed in VLCADD patients, such as non-fluctuating weakness and isolated hyper-CKemia [14]. Furthermore, the severity of these complications can vary greatly among patients with VLCAAD, with some having mild symptoms, while others experience more severe manifestations. Interestingly, there have been reports of individuals suspected to have VLCADD who are clinically asymptomatic [14], which poses a significant challenge when accounting for the incidence of VLCADD. Additionally, the incidence of VLCADD varies in different ethnicities. For instance, Germany reported an incidence of 1:76,000, Taiwan had the lowest worldwide at 1:1,392,000, and Korea had a VLCADD incidence of 1:383,000 [15]. In Saudi Arabia, the incidence of VLCADD is among the world’s highest, reported at 1:37,000 due to the high rate of consanguineous marriages [16]. The incidence of VLCADD may be underestimated for several reasons. Firstly, asymptomatic individuals have not been taken into consideration. Secondly, some VLCADD patients may not have undergone a proper diagnosis. Lastly, there is a possibility of false positive or false negative diagnoses. These factors can significantly impact the accuracy of estimated incidence rates of VLCADD and require appropriate adjustment.

The primary diagnostic approaches for detecting VLCADD are genetic sequencing, encompassing whole genome, or exome sequencing, and the newborn bloodspot screening (NBS) program [17,18]. Genetic testing has limitations due to the variants of uncertain significance (VUS) reported in the *ACADVL* gene [19]. For the NBS program, the measurements of acylcarnitine specifically (C14:1, C14:2, C14, and C12:1) and ratios of acylcarnitine levels (C14:1/C2, C14:1/C16) on dried blood spots are primarily used for VLCADD diagnosis [12].

The increased use of NBS has positively impacted the identification of VLCADD. However, it also poses some drawbacks, such as false negative or false positive results, which may affect the accuracy of the diagnosis [20,21,22,23]. For example, some newborns with severe body weight loss were diagnosed with VLCADD in the first NBS results. However, they were false positive results, and the elevation of C14:1-carnitine was due to the increased catalytic process of fatty acids [24]. Also, it was found that the acylcarnitine profile used for VLCADD patients might be interfering with LCADD [25]. In addition, it was reported that the plasma acylcarnitine profile of carnitine/acylcarnitine translocase (CACT) deficiency disease showed a marked increase in long-chain acylcarnitines and decreased levels of free carnitine [26], which may conflict with VLCADD. Furthermore, the acylcarnitine profile of VLCADD exhibits a low level of carnitine, but this observation has been identified in other disorders related to carnitine transport and cycle [27]. Given the above limitations of the current diagnostic methods for VLCADD, there have been increasing recalls and demands from the clinical field to develop other accurate, alternative diagnostic approaches for the diagnosis and prognosis of VLCADD so that it can be managed and treated with improved accuracy.

Metabolomics, the technique of studying the levels of small molecular weight molecules identified in biological samples such as blood, urine, and dried blood spot (DBS) cards, has been proposed for clinical practice and use as a screening and diagnostic tool for IMDs [28,29,30,31,32]. Since VLCADD is one of the IMDs associated with altered energetic homeostasis and defective metabolism, untargeted metabolomics can be a useful tool to comprehensively investigate the metabolic alterations and mechanisms in VLCADD. Very few studies have focused on applying the metabolomics approach to VLCADD diagnosis [33,34,35]. For example, Miller et al. (2015) applied untargeted metabolomics analyses on plasma samples collected from VLCADD-diagnosed patients to identify distinctive metabolic profiling and biomarkers for VLCADD. Although the number of plasma samples from VLCADD patients in their study was low, their analyses showed interesting metabolic analytes, including myristoylcarnitine (C14), stearoylcarnitine (C18), palmitoylcarnitine (C16), and oleoylcarnitine (C18:1) [33]. Furthermore, a recent study by Knottnerus et al. (2020) aimed to identify metabolic patterns used to distinguish between VLCADD patients with mild or severe phenotypes using untargeted metabolomics. Based on the metabolic results of those patients, they illustrated that the level of C18:2- and C20:0-carnitine, 13,14-dihydroretinol, and deoxycytidine monophosphate were distinctive between mild or severe phenotypic VLCADD patients [34]. Along the same line, in this study, we aimed to identify and uncover distinct metabolic biomarkers and pathways altered in VLCADD patients during the neonatal stage, which could potentially be used as predictive diagnostic biomarkers in early life. Thus, we comprehensively explored the metabolic alterations in VLCADD newborns by performing untargeted metabolomics analyses of DBS collected from newborns diagnosed with VLCADD and healthy newborns.

## 2. Materials and Methods

### 2.1. Ethics Approval

The Institutional Review Boards at King Faisal Specialist Hospital and Research Centre (KFSHRC) in Riyadh, Saudi Arabia (RAC# 2160 027) reviewed and approved this study and its related procedures. In agreement with KFSHRC’s institutional and national legislation, the legal guardians of the VLCADD patients approved the possible use of their banked DBS samples for experimental development and validation.

### 2.2. Patient Inclusion and DBS Collection

**Scheme 1:**
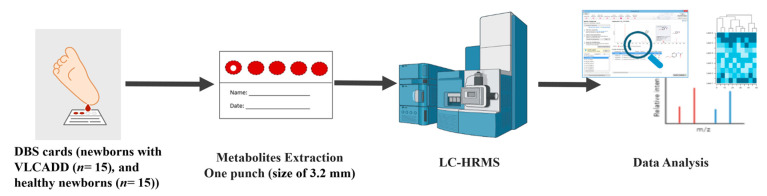
The workflow of the study sample collection, processing, LC-HRMS questioning, and data analysis.

DBS cards used in this study were collected from the metabolomics section in the Center for Genomic Medicine at KFSHRC. Thirty DBS cards included in this study were collected from genetically and biochemically confirmed VLCADD newborns (*n =* 15) and healthy controls (*n =* 15). These healthy controls were age- and gender-matched with the patient group (Scheme 1). The inclusion criteria of this study were applied to the following cases. Firstly, VLCADD patients were only diagnosed with VLCADD. Secondly, the age of participants was a month at maximum. Any study participants not fitting the inclusion criteria were excluded from the study. DBS cards were prepared by dripping blood samples collected from VLCADD and healthy newborns on filter paper called Whatman ProteinSaver 903 using the heel prick method. After that, the DBS cards were dried before storing them in a sealed bag at 4 °C, pending further metabolomics analysis.

### 2.3. Chemicals and Materials

LC-MS-grade acetonitrile (ACN), methanol, formic acid, and water were purchased from Fisher Scientific (Ottawa, ON, Canada).

### 2.4. Sample Preparation

The metabolites were extracted as reported previously with modifications (Scheme 1) [32]. In detail, one punch, a size of 3.2 mm, was collected from each DBS sample and transferred into a 96-well plate for metabolite extraction. Metabolite extraction was performed by adding 250 μL extraction solvent (20:40:40) (H_2_O:ACN:MeOH) to each well with agitation for 2 h at room temperature. Subsequently, sample extracts were dried using SpeedVac (Thermo Fischer, Christ, Germany). The dried samples were reconstituted in 100 μL of 50% A:B mobile phase. (A: 0.1% Formic acid in H_2_O, B: 0.1% FA in 50% ACN:MeOH). Additional punches were collected for quality control (QC) samples and pooled from the study samples to maintain the instrument performance. All study and quality control samples were randomized and placed on the UPLC-QToF-MS autosampler for metabolomics analyses. A quality control sample was analyzed after each set of 5 study samples.

### 2.5. LC-MS Metabolomics

Metabolomics analysis was explored using the Waters Acquity UPLC system coupled with a Xevo G2-S QTOF mass spectrometer equipped with an electrospray ionization source (ESI) [32,36]. The extracted metabolites were chromatographed using an ACQUITY UPLC using an XSelect (100 × 2.1 mm 2.5 μm) column (Waters Ltd., Elstree, UK): the mobile phase composed of 0.1% formic acid in H_2_O as solvent A and solvent B consisted of 0.1% formic acid in 50% ACN:MeOH. A gradient elution schedule was run as follows: 0–16 min 95–5% A, 16–19 min 5% A, 19–20 min 5–95% A, and 20–22 min 5–95% A at a 300 μL/min flow rate. MS spectra were acquired separately under positive and negative electrospray ionization modes (ESI+, ESI−). MS conditions were as follows: source temperature was 150 °C, the desolvation temperature was 500 °C (ESI+) or 140 °C (ESI−), the capillary voltage was 3.20 kV (ESI+) or 3 kV (ESI−), the cone voltage was 40 V, the desolvation gas flow was 800.0 L/h, and the cone gas flow was 50 L/h. The collision energies of low and high functions were set at 0 and 10–50 V, respectively, in MS^E^ mode. The mass spectrometer was calibrated with sodium formate in 100–1200 Da. Data were collected in continuum mode with Masslynx™ V4.1 (Waters Technologies, Milford, MA, USA) workstation. 

### 2.6. Data Processing and Statistical Analyses

The MS raw data were processed following a standard pipeline starting from alignment based on the *m*/*z* value and the ion signals’ retention time, peak picking, and signal filtering based on the peak quality using the Progenesis QI v.3.0 software from Waters (Waters Technologies, Milford, MA, USA) [37]. Features detected in at least 80% of the samples were retained for further analyses. Multivariate statistical analysis was performed using MetaboAnalyst version 5.0 (McGill University, Montreal, QC, Canada) (http://www.metaboanalyst.ca, accessed on 5 January 2023) [38]. For proper selection of the right statistical model, the data sets (compounds and abundances) were mean-normalized, Pareto-scaled, and log-transformed to maintain their normal distribution. The normalized datasets generated principal component analysis (PCA), partial least squares-discriminant analysis (PLS-DA), and orthogonal partial least squares-discriminant analysis (OPLS-DA) models. The OPLS-DA models created were evaluated using the fitness of model (R2Y) and predictive ability (Q2) values using permutation validation of 100 samples. Univariate analysis was performed using Mass Profiler Professional (MPP) software (Agilent Inc., Santa Clara, CA, USA) [39]. Volcano plots were used to identify significantly altered mass features based on a fold change (FC) cut-off of 1.5 and no correction *p* value < 0.05. Venn diagrams were developed using MPP Software. Heatmap analysis for altered features was performed using the distance measure of Pearson. Pathway analysis, biomarkers linked with VLCADD disorder, and receiver operating characteristic (ROC) curves were created using the PLS-DA approach in the MetaboAnalyst v 5.0 for global analysis to identify possible biomarkers.

### 2.7. Peak Annotation (Metabolite Identification)

The significant features in each dataset were selected and tagged in Progenesis QI software for peak annotation. The chemical structures of the metabolites were identified by acquiring their accurate precursor masses, fragmentation patterns, and isotopic distributions for the Human Metabolome Database (HMDB) [40]. The precursor mass and theoretical MS/MS fragmentation tolerance values were set to 5 ppm. The exogenous compounds, such as drugs, food additives, and environmental compounds, were manually excluded from the final list.

## 3. Results

### 3.1. Demographic and Clinical Characteristics of Study Participants

This study had two study groups, including VLCADD and healthy newborns (controls), and their demographic and clinical data are summarized in Table 1. A total of 15 VLCADD and age- and sex-matched 15 healthy newborns were included in the study. The age of the participants in the VLCADD group and healthy control group were 6.2 ± 1.1 days and 5.6 ± 2.5 days, respectively. VLCADD newborns had significantly elevated levels of C14:1-carnitine (2.30 ± 0.51) compared with healthy newborns, as determined by the routine tandem mass spectrometry in the NBS lab. Also, VLCADD newborns showed a significantly increased C14:1/C16-carnitine ratio of 0.44 ± 0.05 when compared with healthy newborns. Thus, we used DBS cards from the participants in the two groups for the subsequent metabolomics analyses.

### 3.2. Metabolomics Profiling of VLCADD Newborns

**Figure 1:**
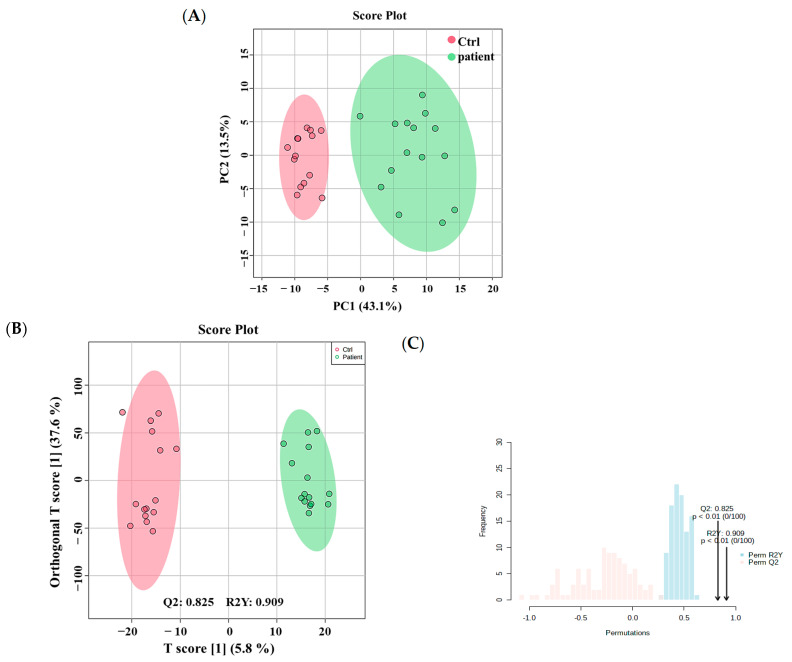
(**A**) Unsupervised principal component analysis (PCA) shows a clear separation between VLCADD newborns and healthy newborns (controls). (**B**) Orthogonal Partial Least Squares—Discriminant Analysis (OPLS-DA) displays a clear separation between the two groups (VLCADD vs. healthy newborn controls). The robustness of the created models was evaluated by the fitness of model (R2Y = 0.909) and predictive ability (Q2 = 0.825) values in a larger dataset (*n* = 100). (**C**) Permutation analysis showing the observed and cross-validated R2Y and Q2 coefficients.

A distinctive metabolomics profile of VLCADD newborns was determined using untargeted analysis based on DBS cards. Metabolomics data showed that 17,542 mass ion features were detected (Table S1), including 11,318 in positive and 6624 in negative ionization modes. To ensure quality in the data analyses, features with missing values >80% were excluded, resulting in 14,593 features remaining for further statistical analysis. Multivariate analysis using unsupervised principal component analysis (PCA) revealed clear clustering and separation between VLCADD newborns (green) and healthy control newborns (red), suggesting there were metabolic changes differentiating these two groups. The total variance of the first two principal components contributed 56.6% in the PCA model for the two study groups (PC1 = 43.1% and PC2 = 13.5%) (Figure 1A). Also, orthogonal partial least squares-discriminant analysis (OPLS-DA) was performed and displayed in (Figure 1B), illustrating group sample clustering and separation between VLCADD and healthy newborns groups. The OPLS-DA model, robust with good predictive ability, was evaluated using permutation analysis with sample number 100 and satisfactory R2Y and Q2 values (R2Y = 0.909 and Q2 = 0.825) (Figure 1C).

**Figure 2:**
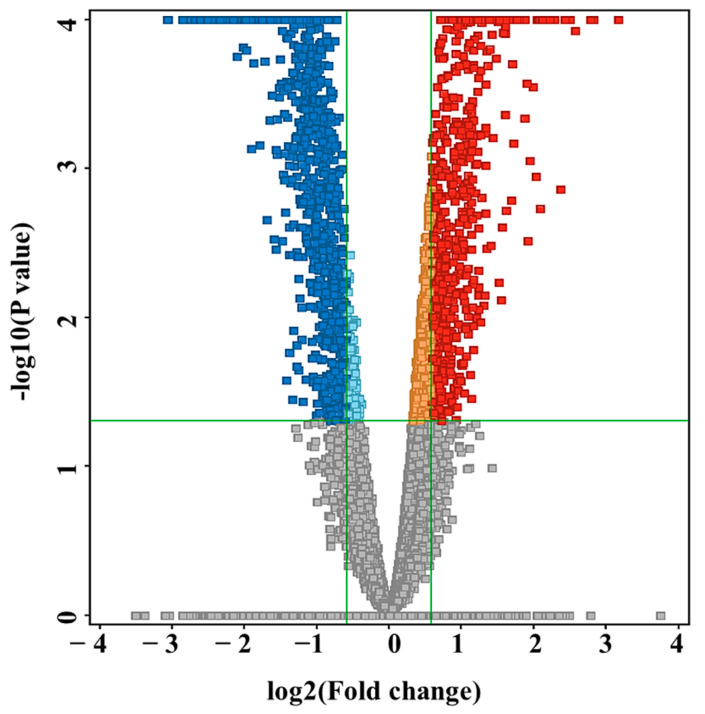
Volcano plot analysis showing 2012 significantly dysregulated metabolites between the two groups. Moderated *t* test, raw *p*-values ≤ 0.05, fold change (FC) 1.5. In VLCADD newborns, 774 metabolites were up-regulated (red) and 1238 were down-regulated (blue).

**Figure 3:**
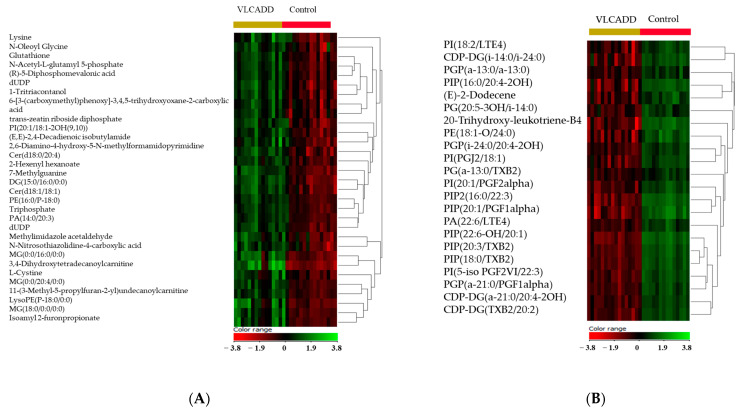
Hierarchal clustering (HAC) and heatmap analysis of the top 206 significantly altered metabolites between the two study groups: VLCADD newborns (gold) and healthy newborn controls (red). (**A**) Heatmap demonstrating up-regulated metabolites and (**B**) down-regulated metabolites in VLCADD newborns compared with healthy newborn controls. The color range bar indicates downregulated metabolites as red and upregulated metabolites as green.

A volcano plot analysis evaluated 14,593 features between the two groups and applied a moderated *t* test, raw *p*-values ≤ 0.05 (*y*-axis), and log_2_(FC) 1.5 (*x*-axis), showing 2012 significantly dysregulated metabolites between the groups (Table S2). In newborns with VLCADD, 774 and 1238 features were up-regulated and down-regulated, respectively (Figure 2). The identification of the 2012 features was conducted with HMDB, resulting in 767 significantly identified metabolites (Table S3). After excluding the exogenous molecules (i.e., drugs, environmental exposures, etc.), 206 metabolites were identified as human endogenous and listed in (Table S4). Fifty-eight significantly upregulated endogenous metabolites were present in VLCADD newborns, as demonstrated in a heatmap (Figure 3A). In contrast, 148 downregulated metabolites were present in VLCADD newborns, and this is partially demonstrated in a heatmap (Figure 3B).

### 3.3. Metabolomic Pathway and Biomarker Analyses

**Figure 4:**
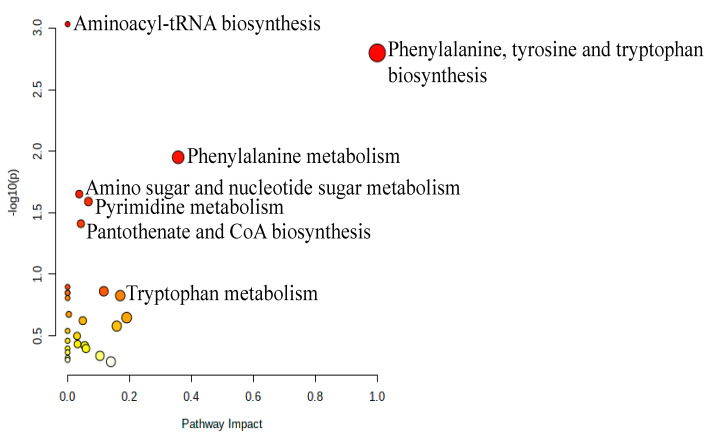
Pathway analysis of significantly dysregulated endogenous metabolites in VLCADD newborns compared to their corresponding healthy newborn controls.

Pathway analysis was performed on the 206 significantly dysregulated endogenous metabolites to identify the most altered pathways in VLCADD newborn patients. Phenylalanine, tyrosine, and tryptophan biosynthesis, aminoacyl-tRNA biosynthesis, amino sugar and nucleotide sugar metabolism, pyrimidine metabolism, and pantothenate and CoA biosynthesis pathways were the most affected in VLCADD, as illustrated in (Figure 4).

**Figure 5:**
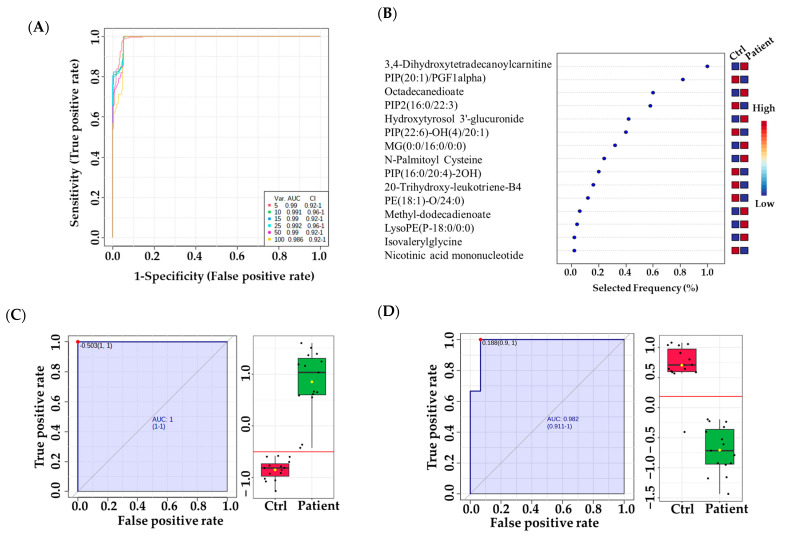

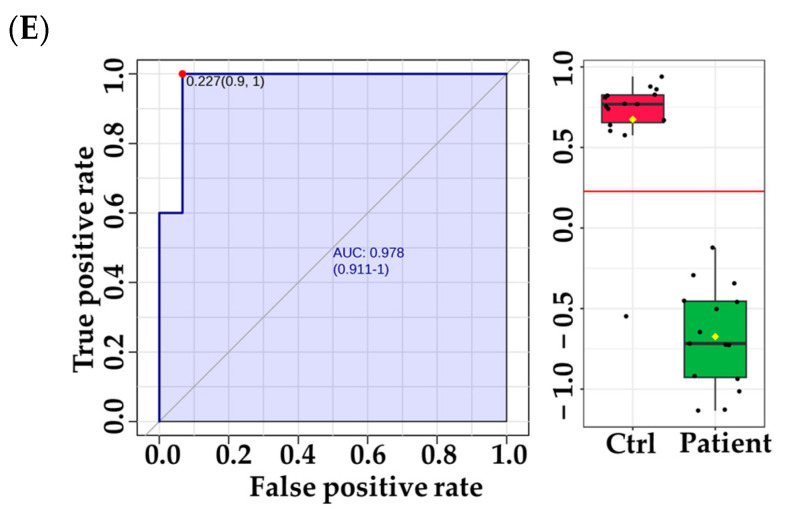
Metabolomics profiling and biomarker evaluation between VLCADD newborns and healthy newborn controls. (**A**) Receiver operating characteristic (ROC) curve for significantly dysregulated metabolites in VLCADD newborns. (**B**) Frequency plot of 15 identified metabolites. (**C**–**E**) Representative ROC curves for significantly dysregulated metabolite molecules in VLCADD newborns. (**C**) 3,4-Dihydroxytetradecanoylcarnitine, AUC = 1; (**D**) PIP (20:1)/PGF1alpha), AUC = 0.982; (**E**) PIP2 (16:0/22:3), AUC = 0.978).

There were significantly altered metabolites involved in these pathways, including L-Phenylalanine, L-Lysine, L-Tyrosine, UDP-alpha-D-galactose, and deoxyuridine diphosphate, which were upregulated. In contrast, L-Valine, L-Tryptophan, 6-deoxy-L-galactose, D-Glucosamine, and deoxycytidine monophosphate deoxyuridine triphosphate were downregulated. In order to identify potential metabolic biomarkers used to distinguish between VLCADD newborns and healthy newborns, receiver operating characteristic (ROC) analysis was performed on the significantly dysregulated metabolites. PLS-DA was used as a classification and feature ranking approach to creating a multivariate exploratory ROC analysis (Figure 5A). ROC curves of the top-ranked metabolites illustrated that the area under the curve (AUC) ranged from 0.986 to 0.992, with confidence intervals (CI) of 0.92–1 and 0.96–1 (Figure 5A). The selected frequency plots represent the 15 significant metabolites with the highest VIP scores in the OPLS-DA model according to their level in VLCADD and healthy newborns (Figure 5B). The selected frequency plot shows metabolites, such as 3,4-Dihydroxytetradecanoylcarnitine (AUC = 1), PIP (20:1)/PGF1alpha) (AUC = 0.982), and PIP2 (16:0/22:3) (AUC = 0.978), as shown in (Figure 5C-E).

## 4. Discussion

### 4.1. Untargeted Metabolomics as a Diagnostic Tool for VLCADD Newborns

VLCADD is one of the most inherited disorders of mitochondrial fatty acid *β*-oxidation, and VLCADD can manifest in early life or adulthood. VLCADD is diagnosed based on family health history, phenotypic symptoms, and clinical testing. Phenotypically, VLCADD is associated with various clinical symptoms, ranging from mild to severe signs such as hypoglycemia, hypoketotic, lactic acidemia, hyperammonemia, cardiomyopathy, and rhabdomyolysis. However, there are VLCADD patients who are reportedly asymptomatic [13,14], which may cause difficulties for diagnosis. Currently, there are two diagnostic methods, those being genetic testing and NBS. Both methods have advanced the diagnosis of VLCADD; however, they have demonstrated some pitfalls and limitations, causing uncertainty in diagnoses. For genetic testing, it has been reported that >300 VUSs in the *ACADVL* gene require functional analyses to determine their potential pathogenicity. For NBS analyses, false negative or positive results have been observed [19,24,41,42], causing doubt and requiring further validation and confirmatory techniques to ensure the accuracy of VLCADD diagnosis. Thus, the urgent need for an additional complementary method to diagnose VLCADD has been raised in the clinical field to increase the accuracy of VLCADD diagnosis. Untargeted metabolomics has been proposed as a promising diagnostic tool for various diseases [28,30,33], which may also be useful for diagnosing VLCADD. There are very few recent studies that have used metabolomics analyses of DBS cards and plasma samples collected from VLCADD patients at different ages to identify potential biomarkers, first to diagnose VLCADD [33] and then to discriminate between VLCADD patients with severe phenotypes and those with mild phenotypes early in life [34]. The potential of metabolomics for diagnosing VLCADD is very promising, which may help find undercover and unrecognized biomarkers that could be used with the current acylcarnitine markers to strengthen and increase the accuracy of VLCADD diagnoses. For that reason, it is necessary to perform further metabolomics analyses of VLCADD patients of different ages with different phenotypes.

Herein, we focused on comprehensive metabolomics analyses of VLCADD during early life. The study used untargeted metabolomics analyses of DBS cards collected from VLCADD newborns, showing distinctive metabolic profiling compared to healthy newborns. Also, altered metabolic pathways and interesting metabolic biomarkers were found to be pronounced in VLCADD newborns. Our findings may help diagnose VLCADD early on and validate other diagnostic methods to achieve greater accuracy.

### 4.2. Distinctive Metabolomics Profile of VLCADD Newborns

Performing untargeted metabolomics showed several metabolites contributing more to the differentiation between VLCADD newborns and their corresponding healthy controls. In particular, there are different categories of lipid metabolites affected in VLCADD newborns, including glycerophospholipids (such as PIP, PA, PG, PE, PGP, PC), glycerolipids (such as TG, CDP-DG, DG, MG) and cardiolipin (CL). It is expected that in the condition of VLCADD, there are many defects in the mitochondrial oxidation of fatty acids, potentially contributing to altercations in other lipid classes that mainly depend on the use of fatty acids in their compositions and concentrations. Our findings are consistent with a recent study that used fibroblasts from VLCADD patients and investigated the lipid signatures of these cells. Their results showed that VLCADD fibroblasts had altered CL, PC, LP, and TG [43].

In addition, gangliosides were more affected in VLCADD newborns compared to their corresponding controls. Gangliosides are sialic acid-containing glycosphingolipids, containing a sphingoid base and sugar residues, and they are involved in maintaining the integration of cellular membranes by controlling the lipid rafts [44,45]. Expectedly, changes in the level or composition of gangliosides negatively impact the integrity of the cell membranes and their modes of interaction with biological molecules in the cells, impacting the overall cellular function.

Not surprisingly, glutathione was elevated in VLCADD newborns, suggesting oxidative stress events resulted from the pathology of the diseases. Glutathione is a cellular tripeptide antioxidant molecule involved in the defense of oxidative stress. Glutathione can mitigate oxidative stress through the detoxification of free radicals. Also, glutathione helps resist lipid peroxidation [46,47,48]. Of note, it was reported that dysregulation of very long chain acyl-CoA dehydrogenase was coupled with lipid peroxidation [49]. These important facts related to glutathione, very long chain acyl-CoA dehydrogenase, and lipid peroxidation may explain the elevation of glutathione in VLCADD newborns.

The metabolic profiling of DBS cards illustrated that several amino acids were dysregulated in VLCADD newborns. Moreover, metabolic pathway analysis showed that multiple amino acid-related pathways, including phenylalanine, tyrosine, and tryptophan biosynthesis, were altered in VLCADD. Mechanistically, amino acids can be involved in the tricarboxylic acid cycle (TCA) to help produce energy eventually. Briefly, amino acids are catabolized, producing their corresponding TCA antimetabolites through transamination reactions to replenish the TCA intermediate metabolites and to keep the TCA cycle going [50]. TCA links amino acids and fatty acids because they both share in synthesizing TCA metabolite. As for fatty acids, they are catabolized to produce acetyl-CoA, which is the first metabolite feeding into TCA [51]. Possibly, alteration of fatty acid oxidation, as seen in the VLCADD condition, results in the decreased production of acetyl-CoA, which may cause abnormalities in the use of amino acids in TCA, showing an overall impact of VLCADD on the metabolism of fatty acids and amino acids, and this may explain the altered amino acid findings in VLCADD newborns. Further research is required to study the cellular modes and mechanisms that impact the levels of amino acids in VLCADD conditions.

### 4.3. Distinctive Metabolic Biomarkers for VLCADD Newborns

The levels of acylcarnitines in fatty acid oxidation disorders, particularly in VLCAAD, are known to be altered due to defects in fatty acid oxidation [52]. Thus, they are used as biochemical diagnostic tests for VLCADD patients. In the VLCADD condition, acylcarnitines and their ratios, specifically C14:1-carnitine, C14:2-carnitine, C14:1/C16-carnitine ratio, C14:1/C2-carnitine ratio, and C14:1/C12:1 ratio, are known to be elevated and used as VLCADD biomarkers [4,5]. Currently, more than one thousand acylcarnitine species have been found, and acylcarnitines are classified based on the length of carbon chains and their acyl moieties’ saturation level and chemical structure [52]; therefore, unrecognized derivatives of acylcarnitines may be correlated with VLCADD. For the first time, our metabolomics data reveal that altered hydroxylated long-chain acylcarnitines, such as 3-hydroxy-5,8-tetradecadienoylcarnitine and 3,4-dihydroxytetradecanoylcarnitine, are found in VLCADD newborns.

Interestingly, the last metabolite mentioned, 3,4-dihydroxytetradecanoylcarnitine, was shown in the biomarker analysis to be a potential biomarker for VLCADD since it was the first metabolite in the top-15 biomarkers. 3,4-dihydroxytetradecanoylcarnitine can be derived from tetradecenoylcarnitine, as a potential biomarker since it was discovered in our metabolomics study and need for validation studies to be used as a confirmed VLCADD biomarker. Interestingly, our findings of hydroxylated long-chain acylcarnitines are consistent with a recent study indicating that hydroxylated long-chain acylcarnitines could be used as biomarkers for mitochondrial myopathy [53]. Thus, hydroxylated acylcarnitines may be found in the VLCADD newborns in our study, particularly 3,4-dihydroxytetradecanoylcarnitine, which could be used as a biomarker for VLCADD.

Notably, phosphatidylinositol phosphates and their oxidized forms were disrupted in VLCADD newborns, and the biomarker analyses revealed that PIP (20:1)/PGF1alpha) and PIP2 (16:0/22:3) were potential biomarkers. Structurally, phosphatidylinositol phosphates are usually located in the cellular membranes and are involved in cellular signaling, apoptotic processes, and proliferation [54,55]. Alterations in phosphatidylinositol phosphates and their derivatives have been correlated with certain metabolic diseases such as insulin resistance and diabetes [56]. In the condition of VLCADD, fatty acid oxidation is defective. This could be associated with abnormalities of the cellular metabolism and structure, explaining our findings of altered phosphatidylinositol phosphates and their derivatives in VLCADD newborns, either as a result or as a cause of the disease. However, more validation and functional studies are warranted to explore our findings further.

Performing a comprehensive metabolomics profile of VLCADD newborns expanded our knowledge of the pathology of the disease. It revealed new insights into the underlying perturbed molecular mechanisms, metabolic pathways, and their related metabolites corresponding with the disease. However, our study has some limitations that must be considered in future studies. The number of samples included in this study is reasonable, but it is necessary to increase the sample size in various independent study cohorts for validation purposes. Also, other omics studies could be conducted to cover all the metabolic alterations in the VLCADD condition comprehensively.

While utilizing an untargeted metabolomics LC-HRMS analysis on DBS samples offers advantages in terms of sample logistics and preservation, it is essential to acknowledge certain limitations; these include the complexity of DBS samples, potential extraction inefficiencies, matrix effects influencing analyte detection, metabolite stability during the drying process, limitations in metabolite coverage, challenges in data analysis, and the restricted dynamic range of LC-HRMS. Researchers should consider these limitations when interpreting results, ensuring a comprehensive understanding of the scope and implications of their findings. By addressing these constraints, future studies can enhance the reliability and applicability of untargeted metabolomics LC-HRMS analysis on DBS samples [57].

Since we used DBS card samples from VLCADD newborns aged less than a month, external factors such as drugs, physical activity, and diet were excluded; thus, it would be ideal to study the impact of these factors on the metabolic profiling of VLCADD by using biological samples from VLCADD patients of different ages, performing various levels of physical activities, and following up with specific treatments/therapies. Furthermore, it would be great to use other biological samples from VLCADD patients, such as plasma, urine, saliva, or skin biopsies, to help find biological samples that are more suitable and reliable for the diagnosis of VLCADD. By overcoming the limitations mentioned above, the diagnosis of VLCADD at an early stage can be improved, which helps select proper relevant treatments for VLCADD patients, improving the health status of the VLCADD patients.

## 5. Conclusions

This study highlights the significant advantages of employing untargeted metabolomics analyses for the diagnosis of very long-chain acyl-CoA dehydrogenase deficiency (VLCADD). Through comprehensive untargeted metabolomics analyses, we successfully identified distinctive metabolic profiles and biomarkers capable of distinguishing VLCADD newborns from their healthy counterparts. Moreover, our findings revealed perturbed pathways, including tryptophan biosynthesis, aminoacyl-tRNA biosynthesis, amino sugar and nucleotide sugar metabolism, pyrimidine metabolism, and pantothenate and CoA biosynthesis in VLCADD. Notably, specific biomarkers such as 3,4-Dihydroxytetradecanoylcarnitine, PIP (20:1)/PGF1alpha), and PIP2 (16:0/22:3) were identified as potential metabolic biomarkers for accurate VLCADD diagnosis. These discoveries pave the way for targeted interventions and treatments that leverage the aforementioned altered metabolic pathways and biomarkers, enabling early-life diagnosis and more effective management of VLCADD. By harnessing the potential of these findings, we can significantly improve human health outcomes by facilitating timely and precise screening and diagnostic approaches for VLCADD, leading to appropriate interventions and personalized care for affected individuals.

## Figures and Tables

**Table 1:** Demographic and clinical characteristics of VLCADD newborns and controls.

Demographic and Clinical Characteristics		VLCADD Newborns (*n* = 15)		Healthy Newborns (*n* = 15)		*p*-Value
		Mean	SEM	Mean	SEM	
Age (Day)		6.20	1.19	5.66	2.5	0.7518
Female (%)		53.3	NA	53.3	NA	NA
Data	C14:1-carnitine (Cutoff: <0.75 μM)	2.30	0.51	<0.75	NA	0.005 **
	C14:1/C16-carnitine ratio (Cutoff: <0.25 μM)	0.44	0.05	<0.25	NA	0.002 **

For statistical analyses, an unpaired student *t* test was conducted. Data are represented by mean ± SEM. Student’s *t*-test; Two-tailed was used, ** p < 0.01.

## Data Availability

The raw data of this study were deposited to Metabolomics Workbench and can be accessed at (accession number ST002560) on 4 May 2023.

## Supplementary Materials

The following supporting information can be downloaded at: https://www.mdpi.com/article/10.3390/metabo13060725/s1, Table S1: Raw data; Table S2: Binary comparison between VLCADD and healthy newborns; Table S3: Exo- and endogenous metabolites were affected in VLCADD newborns; Table S4: Endogenous metabolites were affected in newborns.
